# Antimicrobial activity and mechanism of action of a novel peptide present in the ecdysis process of centipede *Scolopendra subspinipes subspinipes*

**DOI:** 10.1038/s41598-019-50061-y

**Published:** 2019-09-20

**Authors:** Elisa Chaparro-Aguirre, Paula J. Segura-Ramírez, Flavio L. Alves, Karin A. Riske, Antonio Miranda, Pedro I. Silva Júnior

**Affiliations:** 1 Special Laboratory for Applied Toxinology (LETA), Butantan Institute, São Paulo, 05503-900 Brazil; 2 Biomedical Science Institute, University of São Paulo, São Paulo, 05508-000 Brazil; 3 Department of Biophysics, Federal University of São Paulo, São Paulo, 04044-020 Brazil

**Keywords:** Antimicrobials, Pharmaceutics, Peptides, Antimicrobial responses, Protein sequencing

## Abstract

One of the most important cellular events in arthropods is the moulting of the cuticle (ecdysis). This process allows them to grow until they reach sexual maturity. Nevertheless, during this stage, the animals are highly exposed to pathogens. Consequently, it can be assumed that arthropods counter with an efficient anti-infective strategy that facilitates their survival during ecdysis. Herein, we characterized a novel antimicrobial peptide called Pinipesin, present in the exuviae extract of the centipede *Scolopendra subspinipes subspinipes*. The antimicrobial activity of Pinipesin was tested. The haemolytic activity of the peptide was evaluated and its possible mechanism of action was investigated. Identification was carried out by mass spectrometry analysis. Pinipesin displayed potent antimicrobial effects against different microorganisms and showed low haemolytic effects against human erythrocytes at high concentrations. It has a monoisotopic mass of 1213.57 Da, its sequence exhibited high similarity with some cuticular proteins, and it might act intracellularly by interfering with protein synthesis. Our data suggest that Pinipesin might be part of a prophylactic immune response during the ecdysis process of centipedes. Therefore, it is a promising candidate for the development of non-conventional antibiotics that could help fight infectious diseases and represents an exciting discovery for this taxon.

## Introduction

Arthropods, like other invertebrates, rely heavily on their cuticle, which serves as physical barrier to protect against dehydration and invasion of pathogens. When this barrier is damage, the innate immune machinery of these organisms is activated aiming to recognize, neutralize and eliminate the invading agents^1^. Among these defence mechanisms is the production of antimicrobial peptides (AMPs), molecules that have already been isolated from a great variety of natural sources and have emerged as promising candidates for the control of infectious diseases due to their low resistance rates, potent activity and effective mechanism of action^2^. Most AMPs act by disrupting the bacterial cell membrane; however, intracellular targets have also been reported^3^.

Development and growth of myriapods, as in other arthropods, involve a series of moult processes (ecdysis) during which the old cuticle is digested, a new cuticle is formed, and the remnant is discarded (exuviae)^4^. After moulting, a new, soft, and untanned cuticle, that is vulnerable to injury, haemolymph loss and attack is exposed; consequently, these organisms must rapidly melanize and sclerotize their newly formed cuticle to survive. These periods generate heightened biological stress in these animals’ lifetime due to the increased vulnerability to pathogenic attacks and lesions^5,6^.

Although no AMP has yet been isolated from the cuticle waste obtained during the ecdysis process of an arthropod, antimicrobial activity in the cuticle of crustaceans and insects has been previously reported^7,8^. On the other hand, in Eastern medicine, the body extract powder of a centipede, *Scolopendra subspinipes mutilans*, is known for its ability to reduce symptoms of central nervous system deterioration, apoplexy, tetanus and tuberculosis, among other diseases^9^. Consequently, the presence of AMPs in the body extract of these animals has recently been investigated^10,11^.

In light of this, the aim of the present study was to determine the presence of antimicrobial components in the exuviae extract of *Scolopendra subspinipes subspinipes*, a Brazilian centipede belonging to the order Scolopendromorpha.

## Materials and Methods

### Microbial strains

Fungal and bacterial strains were obtained from various sources. *Escherichia coli* SBS 363 and *Micrococcus luteus* A270 were from the Pasteur Institute (Paris, France). *Pseudomonas aeruginosa* Boston 41501 (ATCC 27853), *Bacillus megaterium* ATCC 10778, *Serratia marcescens* ATCC 4112, *Staphylococcus aureus* ATCC 29213, *Alcaligenes faecalis* ATCC 8750, *Bacillus subtilis* ATCC 6633, *Salmonella* Typhimurinum ATCC 14028 and *S*. Arizonae ATCC 13314 were from the ATCC (Manassas, VA, USA). *Enterobacter cloacae* β-12 and *E*. *coli* D31 were from the Stockholm University (Stockholm, Sweden). *Saccharomyces cerevisiae* PM340 (commercial strain) and *Candida albicans* MDM8 were from the Institute of Biomedical Sciences of the University of São Paulo (São Paulo, Brazil). *C*. *tropicalis* IOC 4560 was from the Oswaldo Cruz Institute (Rio de Janeiro, Brazil). The filamentous fungi *Aspergillus niger* and *Beauveria bassiana* (an entomopathogenic fungus) were isolated from bread and a mummified spider, respectively.

### Animals

Adult specimens of *S*. *subspinipes subspinipes* were collected in peridomestic areas and kept alive at the Special Laboratory for Applied Toxinology (LETA) of the Butantan Institute (São Paulo, Brazil) (see Supplementary Fig. S1).

### Ethics

All experimental protocols were approved and conducted according to guidelines and regulations controlled by the Ethics Committee of the Butantan Institute. Animals were collected under the Permanent License for the Collection of Zoological Material No. 11024-3 provided by the Brazilian Institute of Environment and Renewable Natural Resources (IBAMA) and Special Authorization for Access to Genetic Patrimony No. 001/2008.

### Exuviae extract fractionation and Pinipesin purification

The purification of potential AMPs was performed by macerating the fresh exuviae obtained from an adult specimen, in 2 M acetic acid (Synth, Diadema, Brazil) and following the fractionation protocol previously described^11^. The fraction with antimicrobial activity (Pinipesin) was further purified using a linear gradient from 36% to 50% of acetonitrile (ACN) at a flow rate of 1 mL/min for 60 minutes on an analytical Jupiter® C18 column (Phenomenex International). Peptide purity was confirmed by mass spectrometry and amino acid sequencing.

### Antimicrobial assays

During the purification procedure, the antimicrobial activities of the fractions were monitored by liquid growth inhibition assays against *E*. *coli* SBS 363, *M*. *luteus* A270, *A*. *niger*, and *C*. *albicans* MDM8. Bacteria were cultured in poor nutrient broth (PB) (1.0 g peptone in 100 mL of water containing 86 mM NaCl at pH 7.4; 217 mOsm), and fungi and yeasts were cultured in poor potato dextrose broth (1/2- strength PDB) (1.2 g potato dextrose in 100 mL of water at pH 5.0; 79 mOsm). Determination of antimicrobial activity was performed as previously described^12–14^.

### Pinipesin identification

In order to identify the peptide, it was analysed as previously described^14^.

### Analysis with bioinformatics tools

The resulting sequence of Pinipesin was submitted to searches for regions of local similarity against proteins from several sources registered on the public database provided at the National Center for Biotechnology Information (NCBI) using the Basic Local Alignment Search Tool (BLAST) (http://blast.ncbi.nlm.nih.gov/) as well as the CuticleDB database (http://bioinformatics.biol.uoa.gr/cuticleDB/). The physico-chemical parameters of the sequence were calculated using the ProtParam tool available on the bioinformatics resource portal ExPASy (http://web.expasy.org/protparam/). Finally, the ProFunc web server (http://www.ebi.ac.uk/thornton-srv/databases/profunc/), was used to elucidate the potential primary structure of Pinipesin, and the online I-TASSER server (http://zhanglab.ccmb.med.umich.edu/I-TASSER/) was used to obtain a three-dimensional (3D) image of its secondary structure.

### Peptide synthesis

Synthetic Pinipesin and Acetylated Pinipesin (Pinipesin AC) were synthesized as described previously^11^.

### Circular dichroism (CD) spectroscopy

The far-UV (190–250 nm) CD spectra of the peptides were recorded in a Jasco J810 spectropolarimeter (Jasco Inc., Japan) at a concentration of 0.2 mg/mL and 25 °C, with a cylindrical cell of path length of 0.1 cm. The peptides were solubilized in water and 2,2,2-Trifluoroethanol (TFE)/water 50% v/v. All CD spectra were recorded after accumulation of eight runs and smoothed using a Fast Fourier Transform (FFT) filter to minimize background effects. Data are expressed as the mean residue molar ellipticity (deg cm^2^ dmol^−1^).

### Plasma stability

An aliquot (20 μL) of the aqueous peptide stock solution (10 mg/mL) was added to 1 mL of 25% solution of fresh human blood plasma in water at 37 °C. Aliquots (50 μL) were withdrawn and added to 5 μL of pure trifluoroacetic acid (TFA) at different time intervals, then incubated at 5 °C for 15 min. Following incubation, the resulting mixtures were centrifuged at 3000 *g* for 5 min. The supernatants (20 μL) were injected into an online LC/ESI-MS equipment. Elution was performed using an ACN/TFA linear gradient of 3% to 57% ACN at a flow rate of 0.3 mL/min for 30 min. The remaining peptide was monitored by the corresponding peak area in the chromatogram^15^. Assays were conducted in triplicate.

The human blood sample was obtained from a healthy adult donor at Vital Brazil Hospital in accordance with Ethical Guidelines of the Butantan Institute (protocol CEUAIB No. I-1043/13). Written informed consent was obtained from the donor.

### Minimum inhibitory concentrations (MICs) and minimum bactericidal concentrations (MBCs)

The MIC is defined as the minimum concentration of peptide required to achieve 100% growth inhibition^16^. MICs were determined using the synthetic peptides (Pinipesin and Pinipesin AC) against eight Gram-negative bacterial strains, four Gram-positive bacterial strains, three yeasts strains and two fungal strains, as described above (Table 1). The two peptides were dissolved in ultrapure water at a final concentration of 1 mM. MICs of Pinipesin and Pinipesin AC were determined performing serial dilutions in 96-well sterile plates at a final volume of 100 µL, where 20 µL of the peptide were applied to each well at a serial dilution of two-fold microtiter broth dilution and added to 80 µL of the microbial dilution. The lowest concentration without visible growth following incubation at 30 °C for 18 h was defined as the MIC. MBCs were determined after 96 h of incubation at 30 °C. The lowest concentration with no visible growth was defined as the MBC, indicating 99.5% killing of the original inoculum. Microbial growth was measured by monitoring the increase in optical density at 595 nm using a Victor 3TM 1420 multilabel counter (PerkinElmer, Waltham, MA, USA). Assays were performed in triplicate.

**Table 1:** Antimicrobial activity of Pinipesin and Acetylated Pinipesin (Pinipesin AC).

Microorganism	Pinipesin		Pinipesin AC	
	MIC (µg/mL)	MBC (µg/mL)	MIC (µg/mL)	MBC (µg/mL)
**Gram-negative bacteria**				
*Pseudomonas aeruginosa* ATCC 27853	15.16	15.16	ND	NT
*Alcaligenes faecalis* ATCC 8750	15.16	15.16	ND	NT
*Escherichia coli* D31	15.16	15.16	ND	NT
*Escherichia coli* SBS 363	30.33	30.33	ND	NT
*Serratia marcescens* ATCC 4112	30.33	30.33	ND	NT
*Enterobacter cloacae* β-12	30.33	30.33	ND	NT
*Salmonella* Typhimurinum ATCC 14028	15.16	15.16	ND	NT
*Salmonella* Arizonae ATCC 13314	30.33	30.33	ND	NT
**Gram-positive bacteria**				
*Bacillus subtilis* ATCC 6633	30.33	30.33	ND	NT
*Bacillus megaterium* ATCC 10778	15.16	15.16	ND	NT
*Micrococcus luteus* A270	7.58	15.16	ND	NT
*Staphylococcus aureus* ATCC 29213	7.58	7.58	ND	NT
**Yeasts**				
*Candida albicans* MDM8	30.33	30.33	ND	NT
*Candida tropicalis* IOC 4560	30.33	30.33	ND	NT
*Saccharomyces cerevisiae* PM340	30.33	30.33	ND	NT
**Fungi**				
*Aspergillus niger*	30.33	30.33	ND	NT
*Beauveria bassiana*	30.33	30.33	ND	NT

ND, not detected (activity not detected in the range assayed).

NT, not tested.

The MIC and MBC refer to the concentrations necessary to achieve 100% growth inhibition and bactericidal activity, respectively. The highest concentration tested was 100 µM.

### Kinetics of inactivation

To evaluate the minimum time in which Pinipesin acts on microorganisms, the kill curve assay was adapted from the protocol described by Wang^17^. For this assay, 25 μM of purified peptide were added to 200 μL of suspension of *E*. *coli* SBS 363 or *M*. *luteus* A270 at a concentration of 10^6^ cfu and incubated at 37 °C for 0, 5, 10, 15, 30, 60, 120, 180 and 240 min. Following incubation, 20 μL of 5 mg/mL MTT solution were added and incubated at 37 °C for 20 min. The tubes were then centrifuged at 10,000 *g* for 30 s and the supernatant was removed leaving the formazan crystals at the bottom of the tubes. The crystals were dissolved in 1 mL of isopropanol and this volume was transferred to glass tubes, then 1.5 mL of isopropanol were added to yield a final volume of 2.5 mL. The reading was performed in 96-well plates at 595 nm. As positive control, the same procedure was performed with 20 μL of streptomycin instead of peptide; as negative control 20 μL of ultrapure water were used.

### Haemolytic activity

The haemolytic activity of Pinipesin was assayed using human erythrocytes as previously described^11^. A 3% (v/v) suspension of washed erythrocytes in phosphate-buffered saline (PBS) was incubated with Pinipesin at concentrations ranging from 0.5 µM to 500 µM in a 96-well plate for 3 h at 37 °C with intermittent shaking. Absorbance in the supernatant was measured at 414 nm. The haemolysis percentage was expressed in relation to a 100% lysis control (erythrocytes incubated with 0.1% Triton X-100); PBS was used as a negative control. Assays were conducted in triplicate.

Red blood cells were obtained under the same conditions and from the same donor mentioned in the plasma stability assay section.

### Large unilamellar vesicles (LUVs) preparation

LUVs were prepared from solutions of 1-Palmitoyl-2-oleoyl-sn-glycero-3-phosphocholine (POPC) and 1-Palmitoyl-2-oleoyl-sn-glycero-3-phosphoglycerol (POPG). Phospholipids were purchased from Avanti® Polar Lipids (Alabaster, USA). Lipids (pure POPC and POPC/POPG 1:1 molar ratio) were dissolved in chloroform in a test tube, dried under N_2_ steam and kept under vacuum for 2 h. The lipid films were resuspended in 10 mM HEPES pH 7.4 and mixed vigorously in a vortex. The suspension was then extruded at least 13 times through 100 nm pore polycarbonate filter (35) with an extrusion device from Avanti® Polar Lipids to yield LUVs of 100 nm size.

### Isothermal titration calorimetry (ITC)

For this experiment, a VP-ITC (Microcal, Northampton, USA) microcalorimeter was used. The reference cell was filled with water, the calorimeter cell (1.46 mL) was filled with a peptide solution (50 µM Pinipesin) and the syringe was loaded with a dispersion of LUVs (10 mM lipid). The titration experiment consisted of 10 µL injections every 10 min. A first injection of 1 µL was made and discarded from the analysis. The temperature was set to 25 °C.

### Gel retardation assay

The binding of Pinipesin to genomic DNA of *E*. *coli* SBS 363 was evaluated by gel retardation assay^18^. The DNA extraction was carried out as previously described^19^. Four two-fold increasing amounts of Pinipesin (25 to 200 µM) were incubated for 1 hour at room temperature with 500 ng of genomic DNA. Subsequently, the mixtures were analysed by electrophoresis on a 0.8% agarose gel^20^. As negative control, bacterial DNA was incubated without antibacterial agents; and as positive control, it was incubated with three different concentrations of Sarconesin, an antimicrobial peptide that binds and suppresses bacterial DNA electrophoretic migration.

### Total protein profiling of bacterial cells treated with Pinipesin

*E*. *coli* SBS 363 cells were grown in LB medium and the turbidity of the cell suspension was adjusted to a final concentration of 1 × 10^8^ cfu/mL. After growth, cells were harvested by centrifugation at 4,000 *g* for 10 min at 4 °C, the pellets were washed twice with PBS and 10^8^ cells were resuspended in 1 mL of PB medium for further treatment with different concentrations (25, 50 and 100 µM) of Pinipesin for 12 h at 37 °C. Subsequently, the samples were centrifuged at 13,000 rpm for 3 min at 4 °C and the supernatants were discarded. To the pellets, 225 µL of lysis buffer (25 mM Tris–HCl, pH 7.5; 100 mM NaCl; 2.5 mM EDTA; 20 mM NaF; 1 mM Na_3_VO_4_; 10 mM sodium pyrophosphate; 0.5% Triton X-100; protease inhibitor cocktail) containing 30 mM iodoacetamide (IAM) were added and sonication was performed. The supernatants were collected by centrifugation at 14,000 rpm for 15 min at 4 °C and stored at −20 °C. The total protein profiling was carried out by 12% sodium dodecyl sulfate-polyacrylamide gel electrophoresis (SDS-PAGE). The gel was stained with silver nitrate^21^. As positive control, bacterial cells were incubated with streptomycin; and as negative control, they were incubated without antibacterial agents.

## Results

### Purification of pinipesin from the exuviae of *S. subspinipes subspinipes*

**Figure 1:**
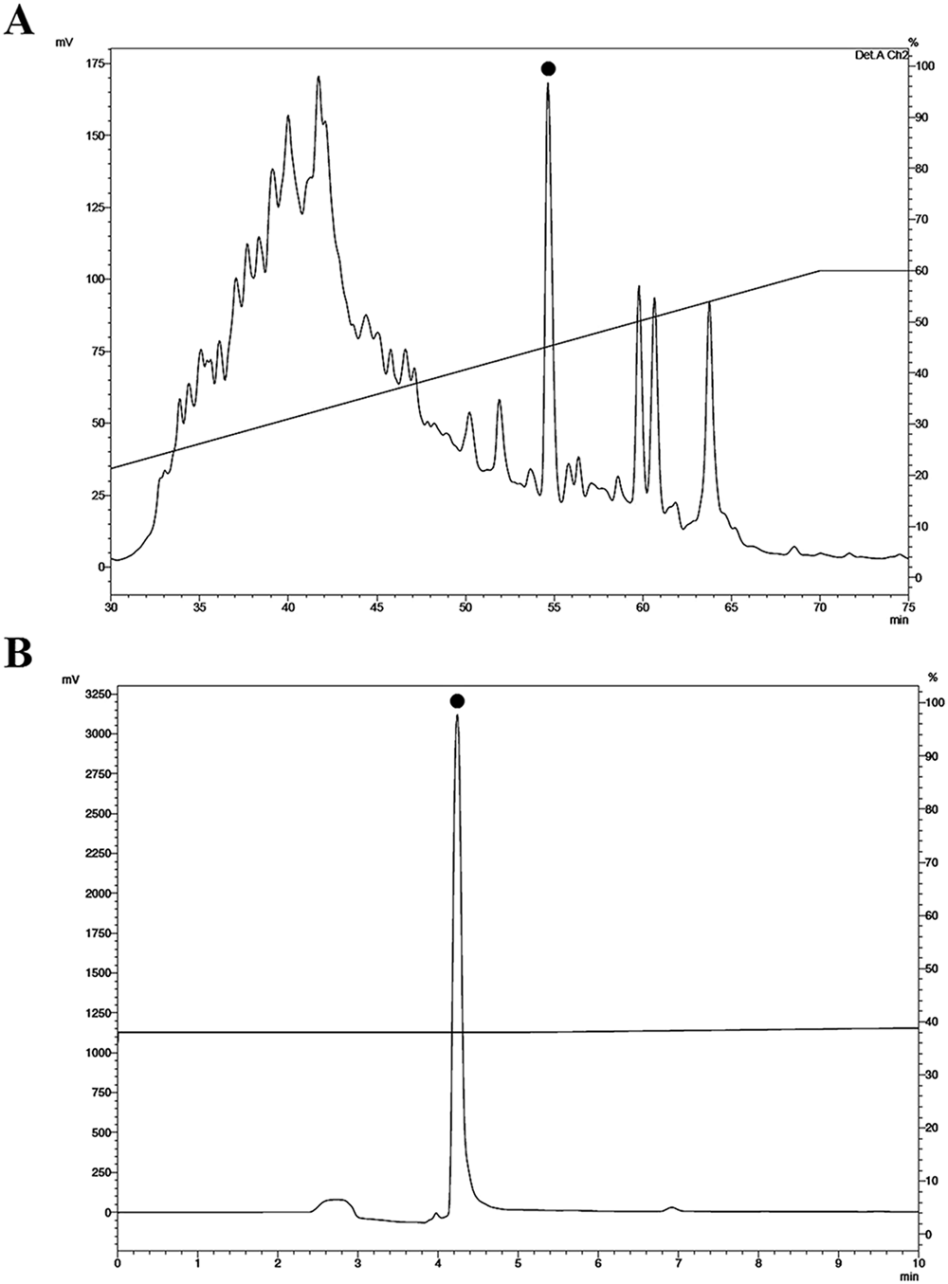
Purification of Pinipesin from the full body extract by reverse-phase HPLC. An acidic extract obtained from *Scolopendra subspinipes subspinipes* exuviae was submitted to solid-phase extraction on Sep-Pak® C18 cartridges. (**A**) The fraction that eluted at 40% acetonitrile (ACN) was analysed on a semi-preparative Jupiter® C18 column with a linear gradient from 2% to 60% ACN in acidified water over 60 min at a flow rate of 1.5 mL/min. A filled circle indicates the only fraction that exhibited antimicrobial activity. (**B**) The fraction with antimicrobial activity was re-chromatographed on the same machine using an analytical Jupiter® C18 column and run from 36% to 50% ACN in acidified water. A filled circle indicates the fraction corresponding to Pinipesin.

The exuviae extract from an adult specimen was processed as previously described. The resulting supernatant was applied to a Sep-Pak® C18 cartridge for pre-purification. Three fractions eluted at 5%, 40% and 80% ACN (with TFA 0.05%) were acquired. The elution at 40% ACN was separated into more than 50 different components by reversed-phase HPLC (Fig. 1A) and all fractions were analysed in liquid growth inhibitory assays using *E*. *coli* SBS 363, *M*. *luteus* A270 and *C*. *albicans* MDM8.

The fraction eluted at 40% ACN was selected for further analysis because previous studies show that several peptides with antimicrobial activity often elute around 40% ACN^11,22,23^. Only one fraction showed antimicrobial activity (Fig. 1A). This fraction was purified (Fig. 1B) and named Pinipesin.

### Sequence determination

**Figure 2:**
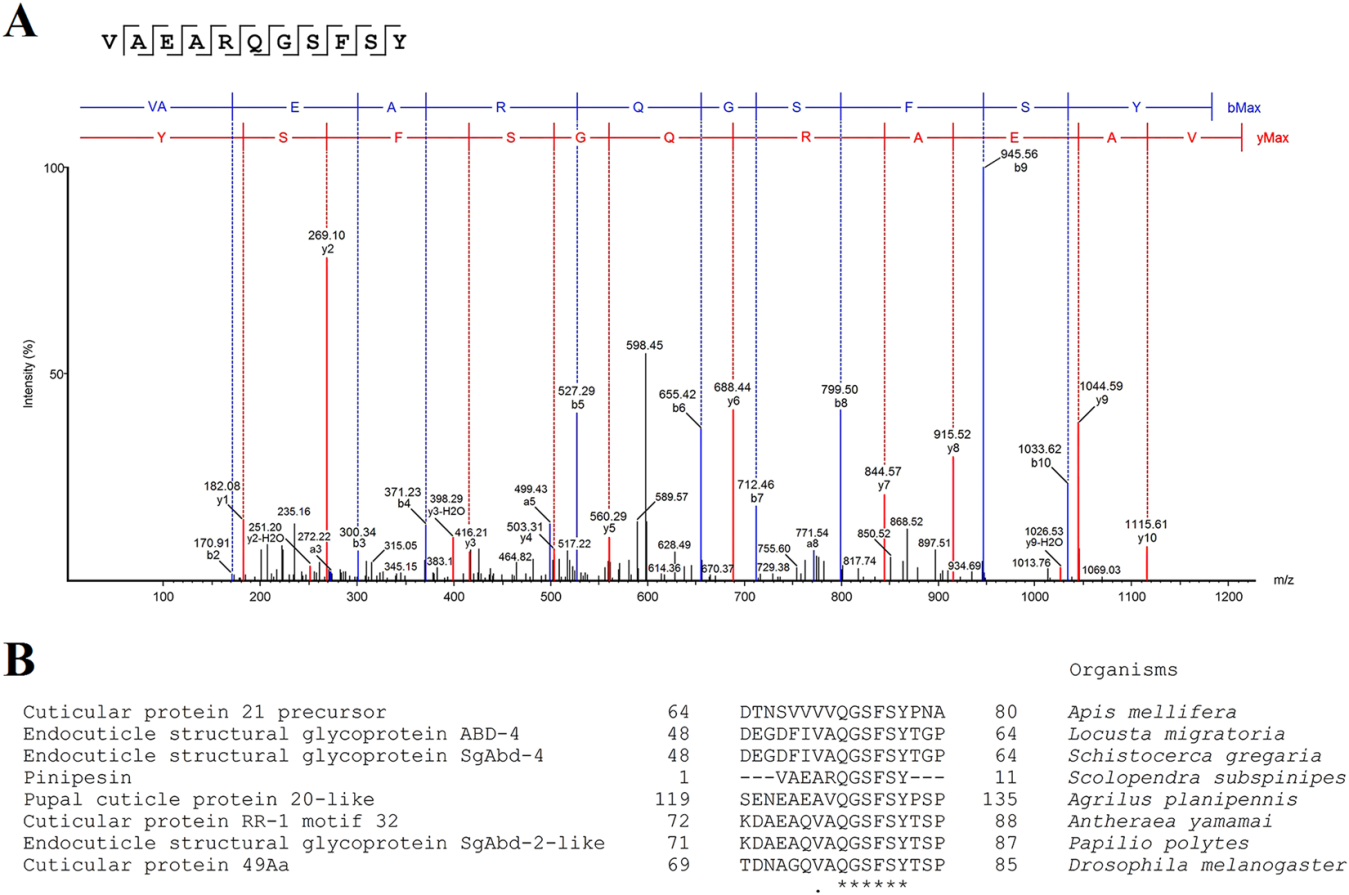
Pinipesin identification. (**A**) Collision-induced dissociation (CID) spectrum of the *de novo* sequenced AMP, Pinipesin. The ions belonging to -y (red) and -b (blue) series indicated at the top of the spectrum correspond to the primary structure of the peptide: VAEARQGSFSY. Internal fragments of the sequenced peptide, whose ions were found in the spectrum, are represented by standard amino acid code letters. (**B**) Multiple alignment analysis of Pinipesin deduced amino acid sequence from *Scolopendra subspinipes subspinipes* with specific fragments of cuticular proteins previously reported from different species of hexapods. Regions that show identical amino acids among all species are indicated by an asterisk (*) and those weakly conserved are indicated by a dot (.). Sequences alignment was performed with Clustal Omega (http://www.ebi.ac.uk/Tools/msa/clustalo/) and modified manually.

Characterization of the Pinipesin primary structure by *de novo* sequencing was performed by analysis of the collision-induced dissociation (CID) spectrum of the peptide using PEAKS Studio software (v7.5). The tandem mass spectrometry (MS/MS) data interpretation revealed a 1213.57 Da sequence of eleven amino acids, VAEARQGSFSY (Fig. 2A). On the other hand, PEAKS DB searches against proteins registered in the Swiss-Prot database did not show a significant match with the *de novo* sequence of Pinipesin, leading us to consider the molecule as a “*de novo* only” peptide, whose resulting average local confidence (ALC) score was 96%.

### Structure and physico-chemical characteristics of Pinipesin

The searches for regions of local similarity between Pinipesin and proteins from arthropods registered on the public database available at the NCBI and the Cuticle DB database showed that all analysed sequences contained a conserved region of six residues (QGSFSY). Additionally, one position was occupied by residues of functional similarity (V and A, both hydrophobic), indicating that the position might represent a conservative amino acid exchange (Fig. 2B).

The theoretical isoelectric point (pI), net charge and molar extinction coefficient (ε) among other peptide properties were calculated (see Supplementary Table S1). Besides, predictions of the primary and secondary structure using the peptide sequence were carried out (see Supplementary Fig. S2).

### CD spectroscopy

**Figure 3:**
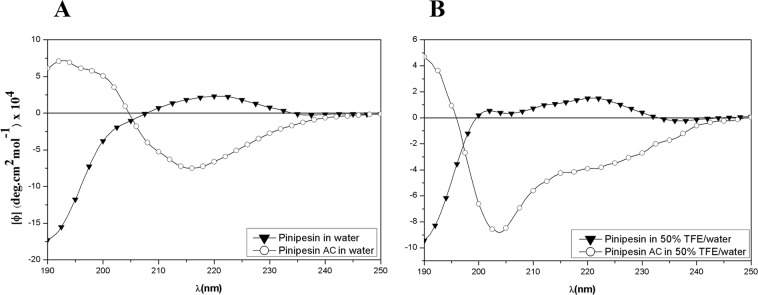
Pinipesin and Acetylated Pinipesin (Pinipesin AC) circular dichroism (CD) spectra. (**A**) Pinipesin and Pinipesin AC CD spectra in water. (**B**) Pinipesin and Pinipesin AC CD spectra in TFE/water 50%. Data are expressed as the mean residue molar ellipticity (deg cm^2^ dmol^−1^).

Secondary structure analysis of Pinipesin was made in a qualitative way, comparing the obtained CD spectra with known secondary structures of the literature^24^. Pinipesin showed a CD profile characteristic of a polyprolin type II (PPII) helix conformation with aromatic contribution (a negative band around 200 nm) in water (Fig. 3A). In the presence of 50% TFE/water, a shoulder rose around 200 nm, indicating a type II β-turn coexisting with the major PPII conformation (Fig. 3B). In water, Pinipesin AC showed a negative band at 218 nm, and a positive one at 196 nm (Fig. 3A). These spectral features are typical of β-sheet conformation. In 50% TFE/water, the CD spectra of Pinipesin AC changed with the negative band becoming stronger and shifting around 203 nm and a positive band around 190 nm (Fig. 3B). These spectral features could be attributed to the coexistence of a major 3_10_ helix and a minor α-helix conformation^25^.

### Plasma stability

**Figure 4:**
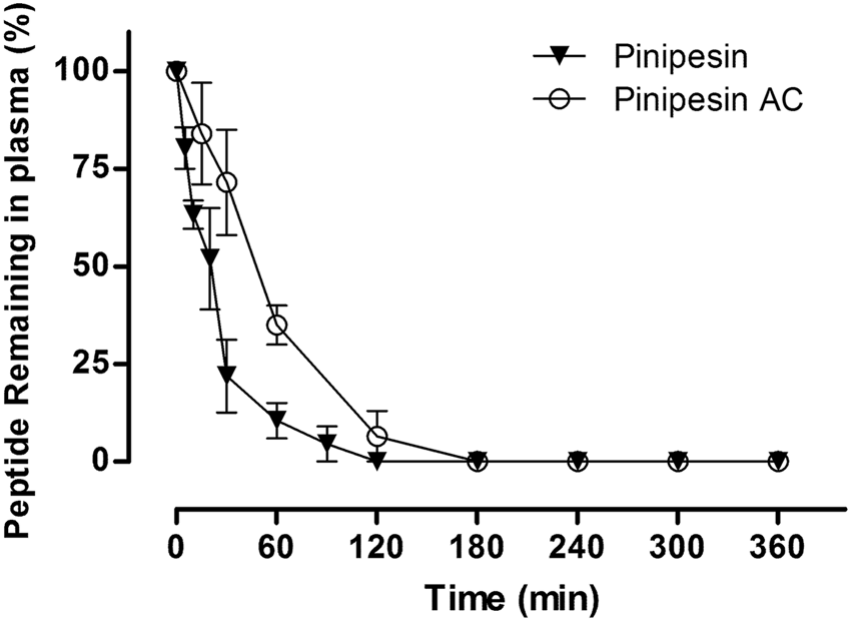
Degradation of Pinipesin and Acetylated Pinipesin (Pinipesin AC) in human plasma over 360 min. Data represent mean percentage of peptide remaining in plasma against time with the error bars representing the standard deviation of three separate experiments.

The plasma stability of Pinipesin and Pinipesin AC was analysed in terms of proteolytic resistance using human blood plasma proteases. As shown in Fig. 4, both were sensitive to proteolysis, being almost totally digested within 60 min. Pinipesin AC stability was expected to be higher than Pinipesin as a result of amidation and acetylation against plasma carboxypeptidases and aminopeptidases. However, the degradation in plasma of both peptides seemed to be comparable. In Pinipesin degradation, hydrolysis occurred with the subsequent elimination of the C-terminal amino acids, indicating the action of carboxypeptidases. In Pinipesin AC degradation, hydrolysis occurred in the middle of the chain, suggesting endopeptidases activity.

### MICs and MBCs

All of the microorganisms tested were sensitive to Pinipesin, which was active at concentrations between 7.58 µg/mL to 30.33 µg/mL. Pinipesin AC did not show antimicrobial activity against any microorganism at the tested concentrations (Table 1).

### Kinetics of inactivation

**Figure 5:**
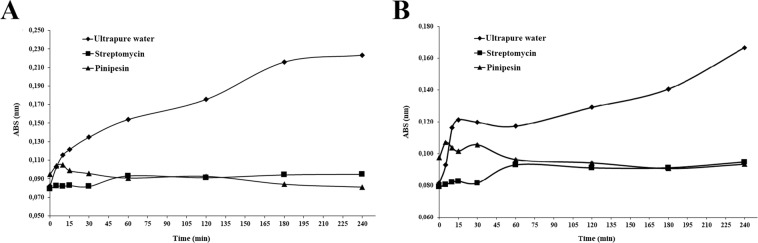
Kinetics of inactivation of *Micrococcus luteus* A270 and *Escherichia coli* SBS 363 by Pinipesin. (**A**) *M*. *luteus* A270 inhibition growth. (**B**) *E*. *coli* SBS 363 inhibition growth. Streptomycin and ultrapure water were used as positive control and negative control, respectively.

When Pinipesin (25 µM) was incubated with *M*. *luteus* A270 or *E*. *coli* SBS 363, the peptide began to inhibit growth after 10 minutes of incubation with the first (Fig. 5A) and 30 minutes with the second (Fig. 5B). In both cases, the peptide reached the same antimicrobial activity as streptomycin after the first hour.

### Haemolytic activity

No haemoglobin release was observed in the concentrations in which Pinipesin was active against bacteria, yeasts and fungi. Nevertheless, at higher concentrations a low percentage of haemolysis was observed (see Supplementary Table S2).

### Peptide-membrane interactions

**Figure 6:**
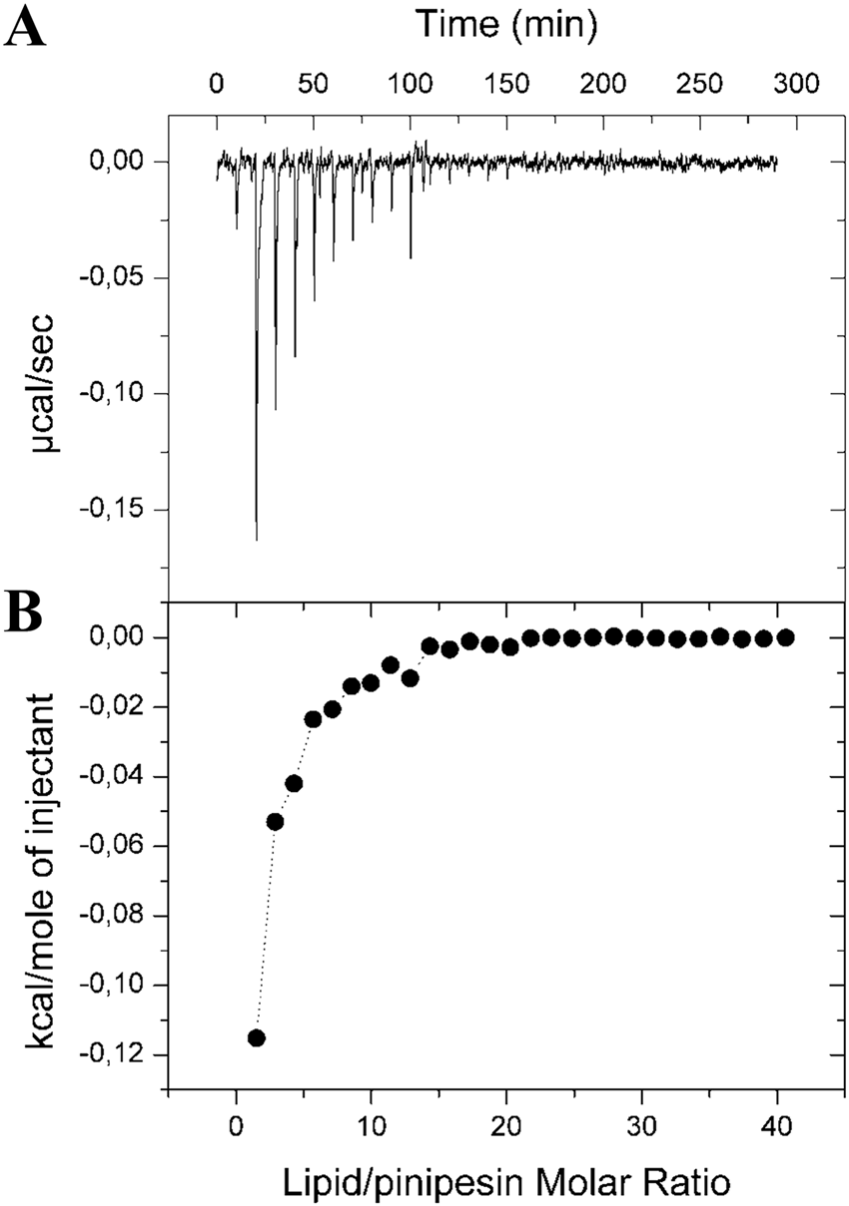
Isothermal Titration Calorimetry (ITC) assay. (**A**) Heat flow. (**B**) Integrated heat per mole of injectant as a function of the lipid/peptide molar ratio.

Titrations of 50 µM Pinipesin with LUVs made of pure POPC and POPC/POPG 1:1 were performed with ITC. No detectable heat was obtained in ITC experiments performed with LUVs made of pure POPC. Figure 6 shows the ITC results of the titration of Pinipesin with LUVs of POPC/POPG 1:1. The interaction of the peptide with anionic LUVs was exothermic and relatively weak (ΔH = −0.4 kcal/mol peptide), as compared with other AMPs^26,27^. Therefore, the antimicrobial activity of Pinipesin might not involve significant peptide-membrane interactions.

### DNA gel movement retardation

**Figure 7:**
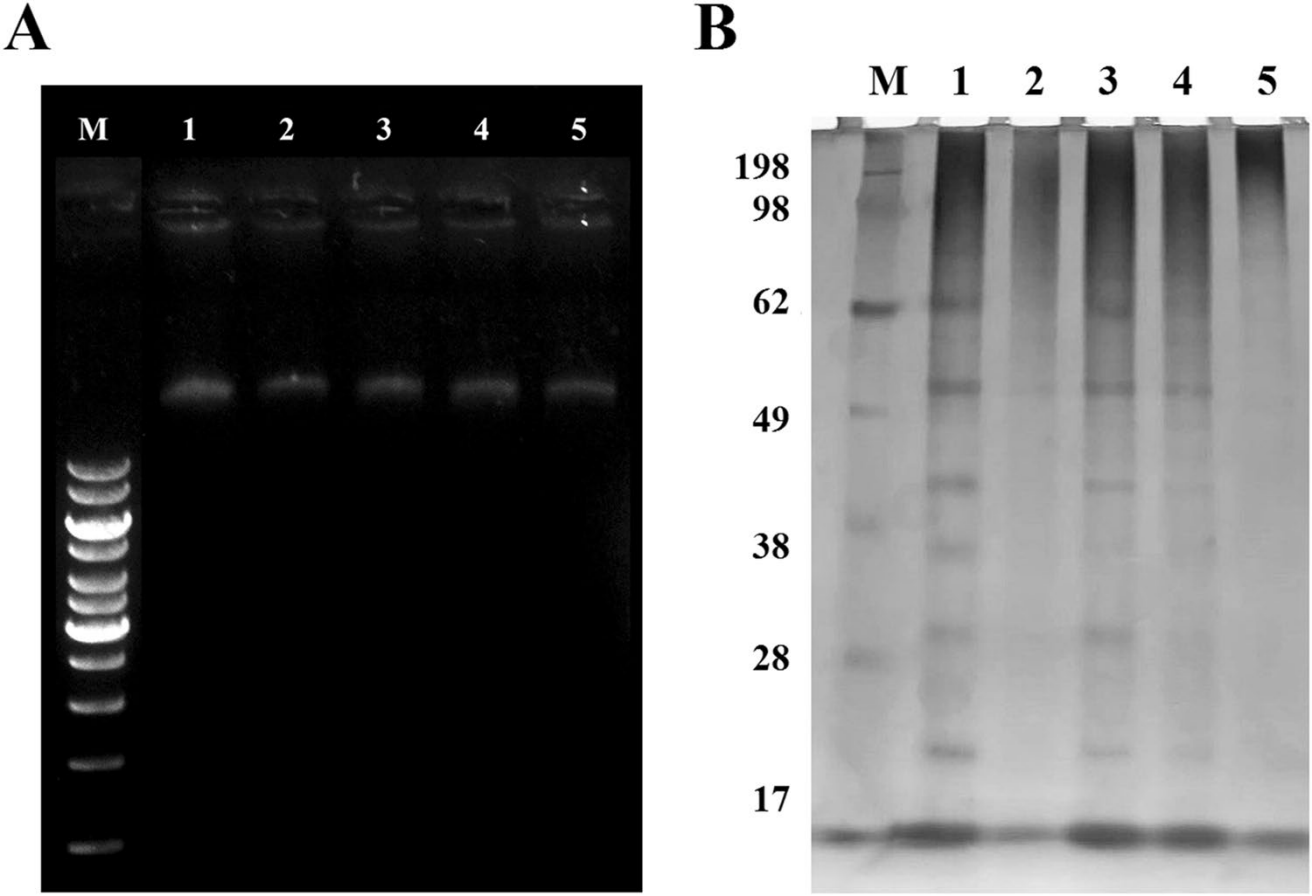
Intracellular targeting mechanism of Pinipesin. (**A**) Analysis of the bacterial DNA-binding activity of Pinipesin by a gel retardation assay. M represents the GeneRuler 1 kb DNA Ladder. 1–5 represent the different Pinipesin concentrations: 0, 25, 50, 100, and 200 µM, respectively. The original agarose gel is shown in Supplementary Fig. S3. (**B**) 12% SDS-PAGE analysis of changes in the protein profile of *Escherichia coli* SBS 363 treated with different concentrations of Pinipesin. M represents the molecular weight marker expressed in kDa. 1 and 2 correspond to the negative and positive control, respectively. 3–5 correspond to the different Pinipesin concentrations tested: 25, 50 and 100 µM, respectively. The original polyacrylamide gel is displayed in Supplementary Fig. S4.

The results of this assay showed that the mobility of DNA was not decreased when the concentration of Pinipesin increased, suggesting that the peptide did not bind to bacterial DNA (Fig. 7A).

### Effects of pinipesin on bacterial protein patterns

To evaluate if Pinipesin has any effect on protein synthesis, the total protein profile of *E*. *coli* SBS 363 was analysed by 12% SDS-PAGE. Numerous clear protein bands were revealed in the negative control, while the protein bands of the bacterium treated with Pinipesin got fainter as the concentration of peptide was increased, and most of them disappeared at the highest concentration (Fig. 7B). The total protein profile treated with 100 µM Pinipesin was significantly similar to the positive control, for which streptomycin, a protein synthesis inhibitor, was used.

## Discussion

Because moulting periods are times of heightened vulnerability to potential injury and attack, production of AMPs during this process represents a form of prophylactic innate immunity that operates to prevent, rather than respond to, infection^28^. Considering this, the resulting multiple sequence alignment lead us to hypothesize that, as has been previously reported^22,29^, Pinipesin might be derived from a specific region of a cuticular protein, a process that might involve a limited proteolysis of the protein. Although there are still no reports of AMPs derived from cuticular proteins in arthropods surfaces^8,30^, recent studies on the functions of moulting fluids produced by insects, revealed that cuticular proteins that were present in moulting fluids, were cleaved and degraded into polypeptide fragments for recycling by several peptidases^31^. This finding indicates that, the resulting peptides from this fragmentation process, might be part of the group of moulting proteins that work together for immune protection during this delicate stage for the animal.

Alternatively, recent studies on shrimp and silkworm moulting have shown that during this stage, the expression of immune-related genes is upregulated^32,33^. As a result, several downstream factors, such as AMPs, are likely to be expressed to generate an immune response during this period. Pinipesin might be one of these expressed effectors that constitute the front line of host defence against infection during moulting. Nonetheless, the true origin of the peptide is still uncertain and remains to be determined in future work.

Pinipesin exhibited activity against Gram-positive bacteria, Gram-negative bacteria, fungi, and yeasts. The observed antimicrobial activity against several drug resistant microorganisms (*E*. *coli* D31 and *S*. Typhimurinum ATCC 14028, resistant against ampicillin and streptomycin; *S*. *aureus* ATCC 29213, resistant to oxacillin and penicillin; and *P*. *aeruginosa* ATCC 27853, resistant against amikacin and piperacillin) was especially interesting^34–37^. Compared to other AMPs isolated from centipedes^11,38–40^, the MICs exhibited by Pinipesin were higher against some bacteria. However, Pinipesin presented a broader spectrum of bioactivity, showing to be effective against all the microorganisms tested, even the entomopathogenic fungi *B*. *bassiana* and *A*. *niger* which have been reported to directly affect the cuticle of arthropods^41^. On the other hand, the absence of bioactivity of Pinipesin AC indicates that the free N-terminal is essential for antimicrobial activity.

The times in which Pinipesin inhibited the growth of bacteria *E*. *coli* SBS 363 and *M*. *luteus* A270 were 10 and 30 minutes, respectively. This is interesting because of the stability of the peptide in human plasma, since the antimicrobial activity of Pinipesin was faster than the rate of degradation of the peptide.

Regardless of the haemolytic activity of the peptide at high concentrations, it is important to mention that even at a concentration five times higher than the highest MIC described, the peptide did not present lytic activity. This makes Pinipesin a potential template for the development of new drugs against microorganism resistant to current medications.

Based on the CD spectra of Pinipesin, and the prediction performed by the I-TASSER server, this molecule apparently does not have a well-defined structure in both hydrophilic and hydrophobic media. Besides, it showed a weak interaction with LUVs and a neutral net charge. This indicates that Pinipesin might not be active against membranes of pathogens^3,42,43^. Consequently, different assays were carried out to determine the possible targets of the peptide. The gel retardation assay showed that Pinipesin did not bind to DNA *in vitro*, suggesting that inhibition of intracellular functions through interference with DNA is unlikely. On the other hand, according to previous reports^44,45^, the reason for gradual disappearance of bands in SDS-PAGE profiles of total bacterial proteins treated with Pinipesin, might be that the peptide interferes with protein synthesis. It is worth noting that the great similarity between the protein pattern treated with 100 µM Pinipesin and the one treated with streptomycin may be due to a common mode of action. In the particular case of the latter, some researches have reported that it distorts the structure of the 30S ribosomal subunit’s decoding site, causing it to incorrectly read the mRNA^46^.

Pinipesin is a new AMP isolated from the exuviae of the Brazilian centipede *S*. *subspinipes subspinipes* that exhibits a broad spectrum of antimicrobial activity and a low cytotoxic activity against human erythrocytes at high concentrations. It is composed by eleven amino acid residues (VAEARQGSFSY), has a monoisotopic mass of 1213.57 Da and an unordered structure. Pinipesin might be part of a prophylactic immune response during the moulting process, a stage of great vulnerability for centipedes. The peptide might interfere with protein synthesis. Further work, though, should be done to fully characterize its mechanism of action.

## Supplementary information

Supplementary Information

## Data Availability

All the data supporting the conclusions have been included within the article.
