# Supplementary material for: Antimicrobial activity and mechanism of action of a novel peptide present in the ecdysis process of centipede Scolopendra subspinipes subspinipes
Source: Sci Rep. 2019 Sep 20;9:13631. doi: 10.1038/s41598-019-50061-y (PMC6754450; doi:10.1038/s41598-019-50061-y)
Supplement: Supplementary file 1 — Supplementary Information [file 41598_2019_50061_MOESM1_ESM.pdf]

Supplementary Material

Title: Antimicrobial activity and mechanism of action of a novel peptide present in the ecdysis process of centipede *Scolopendra subspinipes subspinipes*.

Author List: Elisa Chaparro-Aguirre, Paula J Segura-Ramírez, Flavio L Alves, Karin A Riske,
Antonio Miranda, and Pedro I Silva Júnior.

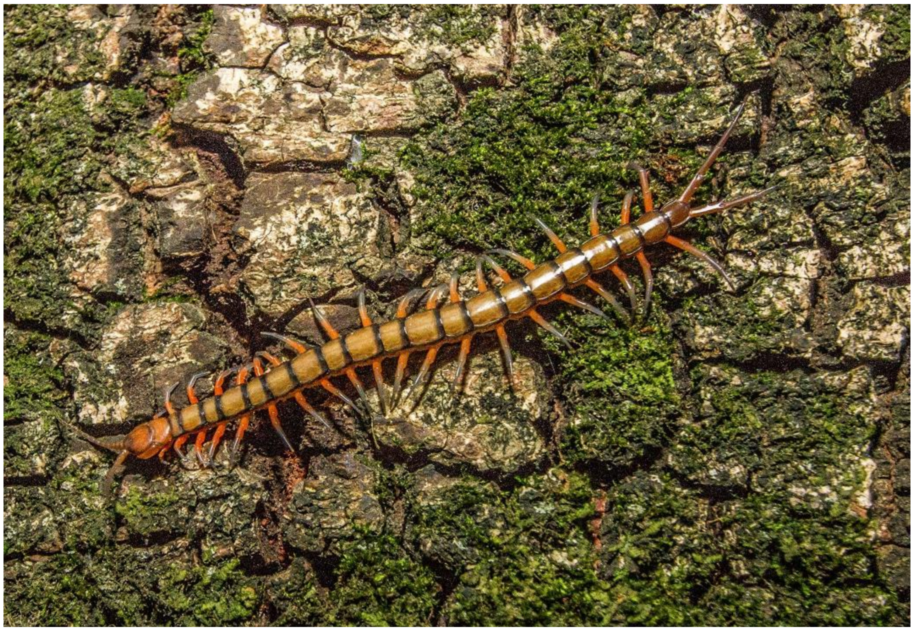

Supplementary Figure S1. *Scolopendra subspinipes subspinipes* (Scolopendromorpha, Chilopoda, Myriapoda). Adult specimens were collected in peridomestic areas and kept alive in the bioterium of the Special Laboratory for Applied Toxinology (LETA) of Butantan Institute.

Supplementary Table S1. Physico-chemical parameters of Pinipesin

Net charge	0
Theoretical isoelectric point (pI)	5.97
Molar extinction coefficient (ε)	1,490 M^{-1} cm^{-1}
Aliphatic index	44.55
GRAVY (grand average of hydropathicity)	-0.382
Instability index	26.60

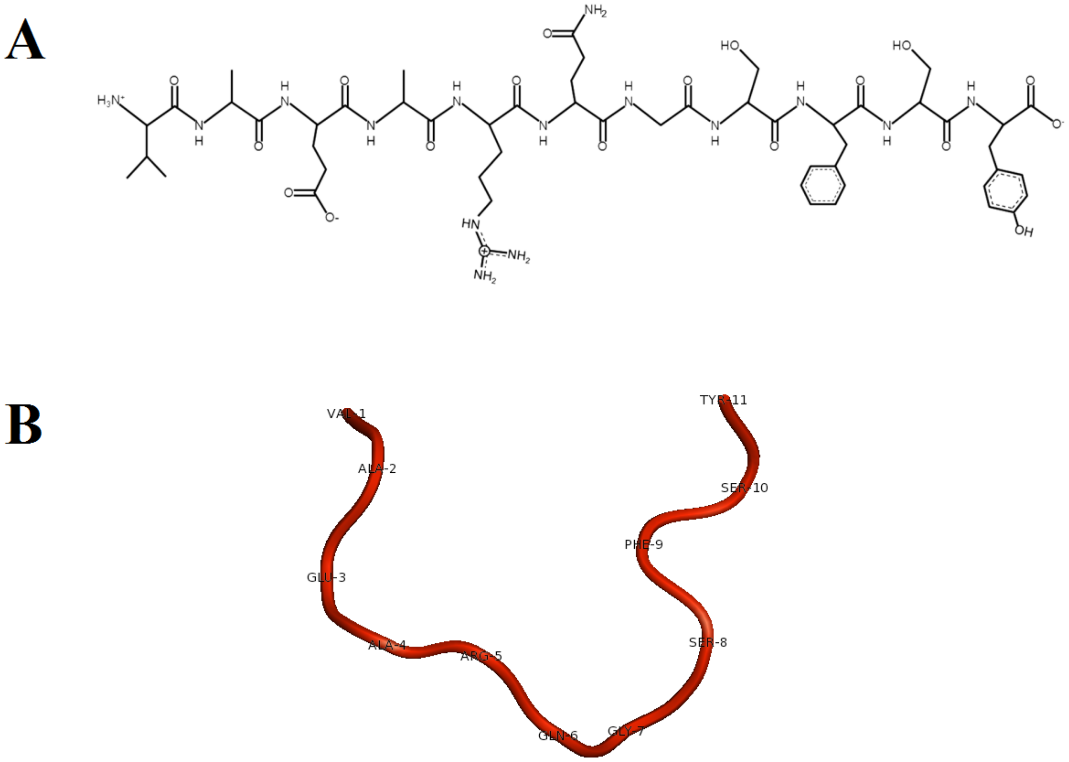

Supplementary Figure S2. Structure Prediction of Pinipesin. (A) Primary structure of Pinipesin. (B) Secondary structure of Pinipesin.

Supplementary Table S2. Haemolytic effects of Pinipesin on human erythrocytes.

Peptide	Haemolysis (%)							
	**1 M**	**500 μM**	**250 μM**	**125 μM**	**32 μM**	**16 μM**	**8 μM**	**4 μM**
Pinipesin	29.12	25.14	0	0	0	0	0	0

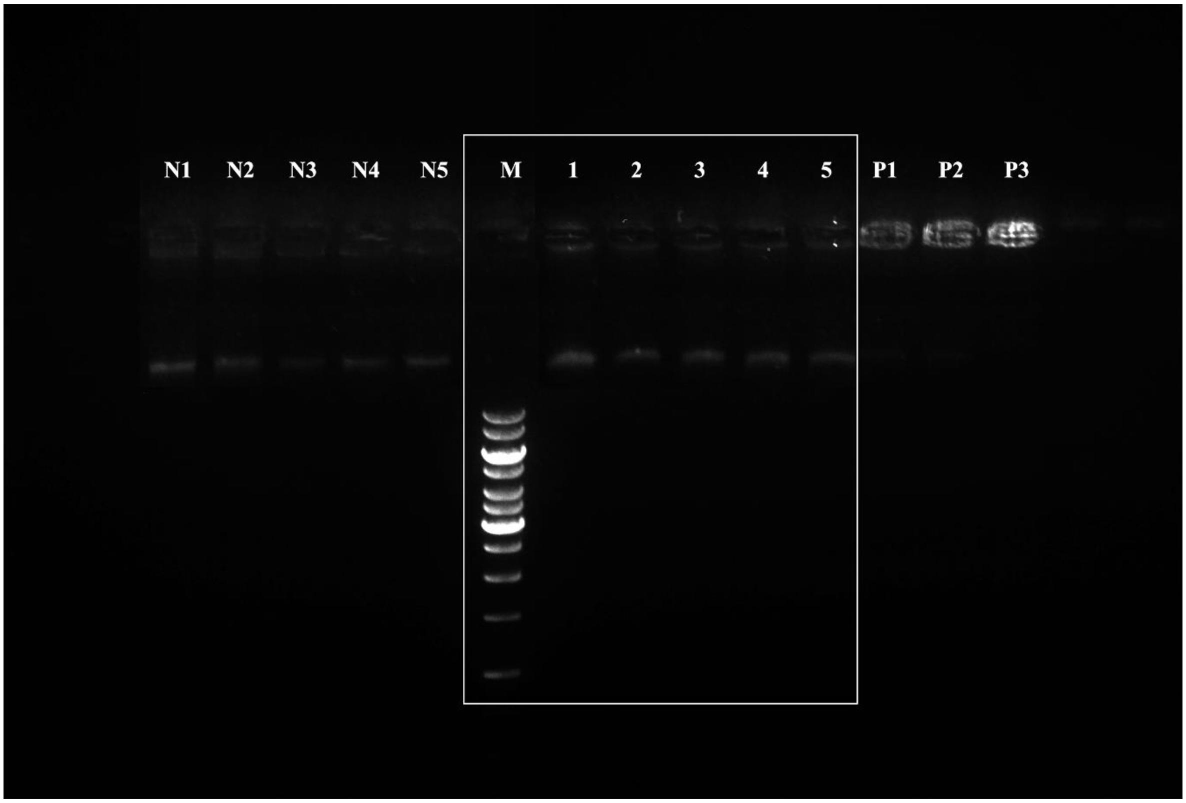

Supplementary Figure S3. Agarose gel of the cropped image shown in Figure 7A. N1-N5 correspond to negative controls, which are five bacterial DNA samples without treatment. P1-P3 represent positive controls, consisting of bacterial DNA treated with three different concentrations of Sarconesin: 50, 100 and 200 µM, respectively.

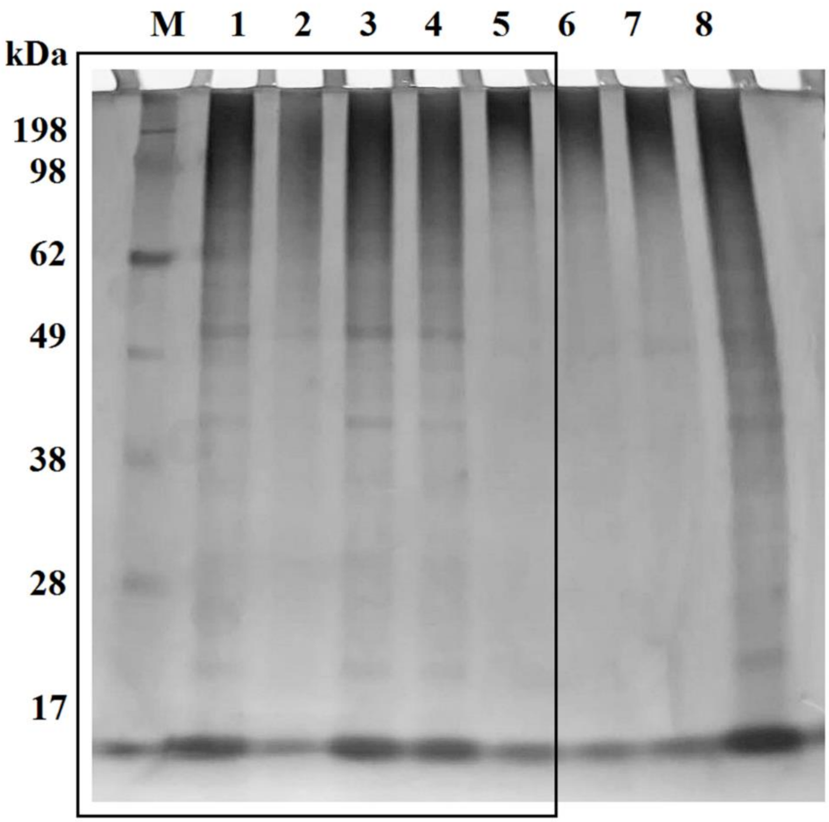

Supplementary Figure S4. Original polyacrylamide gel of the cropped image shown in Figure 7B. 6 and 7 correspond to samples treated with 200 and 400 µM Pinipesin, respectively. 8 represents a second negative control.
